# Evaluation of Nurse‐Led Triage in the Emergency Department: A Retrospective Observational Study

**DOI:** 10.1111/jocn.70320

**Published:** 2026-04-03

**Authors:** Dolores Selva‐Medrano, Jose Alberto Laredo‐Aguilera, Víctor Serrano‐Fernández, Juan Manuel Carmona‐Torres, Ángel López‐González, Inmaculada Pérez‐Rodríguez, Carmen María Guerrero‐Agenjo, Juan David Fernández‐Sánchez, Gloria Mota‐Cátedra, Joseba Rabanales‐Sotos

**Affiliations:** ^1^ Gerencia de Atención Integrada de Albacete, Servicio de Salud de Castilla‐La Mancha (SESCAM) Albacete Spain; ^2^ Grupo de Actividades Preventivas en el ámbito Universitario de Ciencias de la Salud (GAP‐CS) Universidad de Castilla‐La Mancha (UCLM) Albacete Spain; ^3^ Facultad de Fisioterapia y Enfermería Universidad de Castilla‐La Mancha (UCLM) Toledo Spain; ^4^ Grupo de Investigación Multidisciplinar en Cuidados (IMCU) Universidad de Castilla‐La Mancha (UCLM) Toledo Spain; ^5^ Departamento de Enfermería, Facultad de Medicina Universidad Autónoma de Madrid (UAM) Madrid Spain; ^6^ Facultad de Enfermería Universidad de Castilla‐La Mancha (UCLM) Albacete Spain; ^7^ Gerencia de Atención Integrada de Almansa, Servicio de Salud de Castilla‐La Mancha (SESCAM) Albacete Spain; ^8^ Hospital Universitario de Toledo Toledo Spain

## Abstract

**Aim:**

To assess the quality of the Spanish Triage System performed by nurses according to the triage code assigned to each patient and to examine factors associated with the need for re‐evaluation after completion of triage.

**Design:**

Retrospective longitudinal observational study.

**Methods:**

A retrospective analysis was conducted of patients triaged in the emergency department between 2018 and 2023. Patients triaged by other healthcare professionals and those who did not receive a triage priority level were excluded.

**Results:**

493,211 episodes were analysed. Most were low/intermediate acuity (Level IV 65.4%, Level III 23.9%; Level I 0.1%). Mean time‐to‐first physician record entry increased as acuity decreased (38 min Level I vs. 81 min Level V), yet recorded time‐target compliance was lowest in Levels I–II (23.8% and 14.7%). Re‐evaluation occurred more often in high‐acuity levels and was independently associated with older age, male sex, lower oxygen saturation and longer emergency department length of stay; compared with Level I, Levels II–III and lower adjusted odds of re‐evaluation.

**Conclusion:**

Nurse‐led triage demonstrated coherent clinical and operational stratification; however, the lowest recorded time‐target compliance in the sickest patients suggests a gap between immediate care and electronic documentation.

**Implications for the Profession and/or Patient Care:**

Streamline documentation workflows for high‐acuity cases and use re‐evaluation risk profiles to prioritize monitoring and escalation.

**Impact:**

Evidence on nurse‐led Spanish Triage System performance and time‐documentation quality is limited. Acuity and flow metrics showed expected gradients, but target‐time compliance was lowest in Levels I–II; predictors of re‐evaluation were also identified. Findings support emergency department nursing, quality improvement and potential benefits for patients attending emergency departments.

**Reporting Method:**

STROBE guidelines.

**Patient or Public Contribution:**

This study did not include patient or public involvement in its design, conduct or reporting.

## What Does This Paper Contribute to the Wider Global Clinical Community?

- Provides a large benchmark of nurse‐led structured triage over 6 years and 493,211 emergency department episodes, describing how acuity levels map onto vital‐sign documentation, triage duration, time‐to‐first physician record entry and emergency department length of stay.
- Identifies a critical documentation and measurement issue: despite shorter recorded time‐to‐physician entry in high‐acuity patients, target‐time compliance was lowest in Levels I–II, likely reflecting that immediate care may occur before electronic documentation.
- Defines a pragmatic risk profile for physician re‐evaluation, showing a higher likelihood with older age, male sex, lower oxygen saturation and longer length of stay, supporting targeted monitoring and operational prioritization for clinically complex patients across emergency departments.

## 1 Introduction

Triage is the first step in assessing patients when they arrive at the emergency department (ED) (Ausserhofer et al. 2021). During this stage, healthcare staff must determine each patient's level of urgency based on a structured clinical evaluation (Ausserhofer et al. 2021; Smith et al. 2022). This assessment draws on the patient's medical history, vital signs, presenting symptoms and any confirmed or suspected conditions that may indicate physiological instability or risk of rapid deterioration (Ashcraft et al. 2021).

Abdominal pain, nausea, vomiting, chest discomfort, shortness of breath, fever, headache and musculoskeletal complaints are among the most common reasons for seeking emergency care (Giannouchos et al. 2024; Vashi et al. 2019). However, certain conditions—such as cardiac arrest, septic shock, major haemorrhage or pulmonary embolism—pose a far greater threat to life, with mortality rates that can range from 27.7% to more than 80% depending on the underlying cause (Zabel et al. 2024; Piedmont et al. 2024; Jones et al. 2023; Obradovic et al. 2024). These realities underscore the critical role of nurses during triage: timely and accurate nursing assessments help reduce delays in care and ensure that patients receive interventions aligned with the severity of their condition, ultimately contributing to better outcomes in the ED (Melgar‐Perez et al. 2025; Grant et al. 2020).

Depending on the degree to which the patient's condition may compromise clinical stability, the patient is assigned a priority level (Smith et al. 2022; Tsiftsis et al. 2025; Peta et al. 2023). These levels may vary according to the triage system used (Zaboli et al. 2021; Kalan et al. 2024; Mollet et al. 2023). Nevertheless, across systems, greater instability and higher risk to health or life are associated with shorter target times to initiate care and to receive the first physician assessment (Zaboli et al. 2021; Kalan et al. 2024; Mollet et al. 2023). In this context, multiple triage systems are used worldwide, including the Manchester Triage System (MTS), the Emergency Severity Index (ESI) (Tsiftsis et al. 2025; Ingielewicz et al. 2024), Canadian Triage and Acuity Scale (CTAS) (Kalan et al. 2024; Ingielewicz et al. 2024) or the Australasian Triage Scale (ATS) (Ingielewicz et al. 2024; Raubenheimer et al. 2025). Specifically, the MTS is the reference system in settings such as the United Kingdom and several Western European countries, including Germany and the Netherlands (Peta et al. 2023; Gräff et al. 2014). The ESI is widely used in the United States, whereas CTAS is the predominant triage system in Canada (Koca et al. 2023). In turn, the ATS is the most used triage system in regions such as Australia and New Zealand (Ingielewicz et al. 2024; Raubenheimer et al. 2025).

Although several of these triage systems are also used in Spain, one of the most prominent is the Spanish Triage System (STS) (Font‐Cabrera et al. 2025). This triage model was adopted as a national standard by the Spanish Society of Emergency Medicine and is based on the Andorran Triage Model (Gomez Jimenez 2003). It is a structured, nurse‐led, non‐exclusionary triage system supported by computerized decision‐support tools that assist clinical decision‐making (Font‐Cabrera et al. 2025; Gomez Jimenez 2003). The STS distinguishes five acuity levels according to the patient's clinical severity, risk of death, and need for immediate care. In this framework, acuity ranges from priority Level I, corresponding to life‐threatening emergencies, to priority Level V, considered the least urgent due to the milder characteristics of the patient's condition (Font‐Cabrera et al. 2025). In the STS, priority level is assigned through a structured nurse‐led assessment based on the patient's main presenting complaint. Patients are first classified into a symptom category, after which urgency is determined using specific questions, clinical discriminators, vital signs, pain assessment and severity scales supported by computerized triage decision‐support programme (Soler et al. 2010).

In empirical triage research, performance can be examined across different dimensions, including the coherence between assigned acuity and clinical documentation, the operational consequences of priority assignment within ED flow and the need for subsequent reassessment after triage (Sibbritt et al. 2006; Jiménez et al. 2003; Hinson et al. 2019; Kuriyama et al. 2017). In retrospective real‐world datasets, these dimensions may be captured through pragmatic process and monitoring indicators rather than through a single gold‐standard outcome (Zaboli, Brigo, Sibilio, et al. 2025).

However, evidence regarding triage effectiveness is mixed (Font‐Cabrera et al. 2025; Camilo et al. 2020; García‐Martínez et al. 2025), as some publications identify STS as the preferred triage model among patients (Font‐Cabrera et al. 2025; Camilo et al. 2020), whereas other studies report that, among 470 patients, 150 were incorrectly triaged—approximately 32% (García‐Martínez et al. 2025). Given this and considering the importance of triage for subsequent patient care, it is necessary to evaluate the quality of nursing performance during triage.

Therefore, the aim of this study is to describe the distribution of STS priority levels and to examine their associations with clinical documentation patterns, operational flow indicators and the need for re‐evaluation after triage. In addition, we explored factors associated with the need for re‐evaluation as a post‐triage clinical monitoring indicator.

## 2 Methods

### 2.1 Design

To meet the objectives of this study, we conducted a retrospective longitudinal observational study based on routinely collected hospital data. The analytical scope was primarily descriptive, aimed at characterizing STS priority‐level distribution and associated operational indicators in the ED, and exploratory regarding the analyses of factors associated with re‐evaluation.

This analysis was performed in accordance with the Strengthening the Reporting of Observational Studies in Epidemiology (STROBE) guidelines for observational studies (von Elm et al. 2008).

### 2.2 Study Population

The sample included all individuals who required care in the ED, specifically at triage. Data were collected at Albacete General Hospital between 2018 and 2023.

Patients of any age who required triage and were assessed by nursing staff before entering the ED care pathway were included. Patients triaged by other healthcare professionals or those who were not assigned a priority level were excluded.

### 2.3 Variables

To describe the sample, we analysed the sociodemographic variables sex, age, age groups, and reason for the ED visit.

To assess the quality of nurse‐led triage, we considered the assigned triage priority levels; recorded vital signs including systolic blood pressure (SBP), diastolic blood pressure (DBP), mean arterial pressure (MAP), heart rate (HR) and oxygen saturation; the proportion of vital signs recorded by nurses; time spent in triage; total ED length of stay (LOS); the need for re‐evaluation in the ED; and the number of additional assessments required. Re‐evaluation was calculated based on matching triage IDs within the same clinical episode. Triage performance was examined through operational indicators and the need for re‐evaluation; the latter being interpreted as a clinically relevant post‐triage proxy indicator (Zaboli, Brigo, Sibilio, et al. 2025).

In addition, we analysed the time elapsed between triage completion and the initiation of the medical record by the physician. To determine whether time‐to‐care targets were met, we used the cut‐offs established in the current regional regulation in Castilla‐La Mancha (Decreto 45/2019 de 21 de mayo) (Gobierno de Castilla‐La Mancha 2019).

### 2.4 Procedure

The data analysed in this study were obtained from Albacete General Hospital. The hospital maintains annual databases containing information on patients presenting to the ED. This information was provided to the research team after approval was obtained from the ethics committee.

After data for each year were obtained, the datasets were merged into a single file. The resulting dataset was cleaned and processed using IBM SPSS Statistics version 29 under a licence provided by the University of Castilla‐La Mancha.

### 2.5 Ethical Considerations

This study was approved on May 29, 2025, by the Research Ethics Committee for Medicinal Products at Albacete General Hospital with approval code: 2024‐096. Written informed consent was obtained from participants before their information was included in the hospital database.

All data were analysed in anonymized form, preventing patient identification.

### 2.6 Statistical Analysis

All analyses were performed using IBM SPSS Statistics version 29. Categorical variables were summarized as frequencies (*n*) and percentages (%), whereas continuous variables were described using the arithmetic mean (*m*) and standard deviation (SD). Comparisons between categorical variables were conducted using the Chi‐square test.

Before comparing continuous variables, assumptions of normality and homogeneity of variances were assessed using the Shapiro–Wilk test and Levene's test, complemented by graphical inspection. When assumptions of normality and homoscedasticity were met, one‐way analysis of variance (ANOVA) was applied, with Bonferroni‐adjusted post hoc comparisons. In the presence of heteroscedasticity, one‐way ANOVA with Games‐Howell post hoc comparisons was used. When the normality assumption was not met, the Kruskal–Wallis test was performed.

Subsequently, bivariate correlations between continuous variables were examined using Pearson's correlation coefficient.

Finally, exploratory logistic regression analysis was performed to identify factors associated with the need for physician re‐evaluation in the ED. These analyses were intended to examine associations with a post‐triage clinical indicator. Both univariable (crude) and multivariable (adjusted) logistic regression models were estimated. Variable selection for the multivariable model was conducted using a forward stepwise procedure based on the Wald statistic, and results were reported as odds ratios (OR). Overall model fit for the multivariable model was assessed using the Nagelkerke *R* ^2^.

All hypothesis tests were two‐tailed, with a 95% confidence interval (CI), and statistical significance was set at *p*‐values < 0.05.

## 3 Results

### 3.1 Sample Characteristics

Overall, the database contained information on 530,208 ED presentations. Of these, 36,997 patients were excluded because they were triaged by other healthcare professionals or did not have a priority level assigned according to the STS. Therefore, the final analytical sample comprised 493,211 patients who were assigned to an STS priority level at Albacete General Hospital between 2018 and 2023.

Among the included patients, 51.5% were women and 48.5% were men, with ages ranging from 14 to 96 years. The age group accounting for the highest proportion of ED visits was 18–44 years (34.6% of all visits). The most frequent reasons for ED presentation were trauma‐related conditions (21.4%) and infectious diseases (16.4%), whereas the least frequent triage categories were obstetric‐related conditions (0.4%) and haemorrhage/bleeding (0.5%). Table 1 summarizes the sociodemographic characteristics of the sample.

**TABLE 1:** Sociodemographic characteristics.

Variable	Overall patients (*N* = 493,211) (%)
Sex	
Male	239,310 (48.5)
Female	253,901 (51.5)
Age (mean ± SD)	53.69 ± 22.38
Age groups	
< 18 years	19,685 (4.0)
18–44 years	170,612 (34.6)
45–64 years	135,116 (27.4)
65–74 years	61,360 (12.4)
> 75 years	106,438 (21.6)
Reason for visit	
Trauma	105,757 (21.4)
Digestive problems	60,496 (12.3)
Infectious diseases	81,036 (16.4)
Respiratory diseases	28,873 (5.9)
Cardiovascular diseases	46,291 (9.4)
Neurologic diseases	32,512 (6.6)
Dermatological diseases	17,047 (3.5)
Non‐specific pain	43,343 (8.8)
General malaise	39,128 (7.9)
Haemorrhage/Bleeding	2026 (0.4)
Metabolic diseases	12,219 (2.5)
Mental health	7002 (1.4)
Scheduled visit	14,984 (3.0)
Obstetrics	2497 (0.5)

Abbreviation: SD, standard deviation.

### 3.2 Triage Priority Levels and Vital Signs

The distribution by triage priority level showed that most episodes corresponded to low and moderate urgency, with a small proportion of patients classified at the highest priority level. Specifically, most patients were assigned to Level IV (322,394; 65.4%) and Level III (125,946; 23.9%), followed by Level V (24,968; 5.0%) and Level II (19,656; 4.0%), whereas Level I had the lowest assignment rate (517; 0.1%).

Regarding vital signs, body temperature was slightly higher among patients triaged to Levels II and III (36.71°C ± 0.86°C and 36.64°C ± 0.81°C, respectively) and was lower among those assigned to Level V (36.47°C ± 0.55°C). Blood pressure values were lowest in Level I (SBP 122.91 ± 29.76 mmHg; DBP 70.12 ± 18.33 mmHg; MAP 96.62 ± 22.35 mmHg), increased in Levels III and IV (SBP approximately 136–137 mmHg and MAP 109–110 mmHg), and then decreased slightly in the lowest priority level.

Heart rate was higher in the most urgent categories, reaching the highest mean value in Level II (91.86 ± 23.14 bpm), and remained stable across Levels III–V (85.61 ± 20.70, 84.49 ± 17.42 and 83.18 ± 17.39 bpm, respectively). Table 2 presents vital sign values and statistically significant differences across priority levels.

**TABLE 2:** Vital signs at triage.

Variable	Triage priority level, *n* (%)				
	Level I	Level II	Level III	Level IV	Level V
	517 (0.1)	19,656 (4.0)	125,946 (23.9)	322,394 (65.4)	24,968 (5.0)
Temperature (°C)	36.39 ± 0.80	36.71 ± 0.86^a^	36.64 ± 0.81^a^, ^b^	36.57 ± 0.63^b^, ^c^	36.47 ± 0.55^b^, ^c^, ^d^
SBP (mmHg)	122.91 ± 29.76	127.24 ± 28.06	136.96 ± 28.46^a^, ^b^	136.60 ± 23.09^a^, ^b^, ^c^	130.51 ± 23.68^a^, ^b^, ^c^, ^d^
DBP (mmHg)	70.12 ± 18.33	76.73 ± 16.46^a^	81.17 ± 16.71^a^, ^b^	83.30 ± 13.67^a^, ^b^, ^c^	78.58 ± 13.54^a^, ^b^, ^c^, ^d^
MAP (mmHg)	96.62 ± 22.35	101.94 ± 20.57^a^	109.01 ± 20.65^a^, ^b^	109.91 ± 16.58^a^, ^b^, ^c^	104.42 ± 16.74^a^, ^b^, ^c^, ^d^
Heart rate (bpm)	87.72 ± 23.98	91.86 ± 23.14	85.61 ± 20.70^b^	84.49 ± 17.42^b^, ^c^	83.18 ± 17.39^b^, ^c^, ^d^
Oxygen saturation	95.37 ± 6.09	92.57 ± 7.16^a^	94.00 ± 4.52^b^	96.29 ± 2.67^b^, ^c^	96.18 ± 3.13^b^, ^c^

*Note:* Data are presented as mean ± standard deviation.

Abbreviations: °C, degrees Celsius; BDP, diastolic blood pressure; bpm, beats per minute; MAP, mean arterial pressure; mmHg, millimetres of mercury; SBP, systolic blood pressure.

^a^ *p* < 0.05 versus Level I.

^b^ *p* < 0.05 versus Level II.

^c^ *p* < 0.05 versus Level III.

^d^ *p* < 0.05 versus Level IV (ANOVA post hoc tests).

### 3.3 Care Performance and Clinical Processes Across Triage Levels

Regarding the mean time from triage completion to the first physician entry in the medical record, shorter intervals were observed in the highest acuity categories, with mean values of 38.00 ± 43.07 min and 44.15 ± 49.16 min for Levels I and II, respectively, compared with Level V, where this interval was 80.59 ± 84.80 min. These differences were statistically significant (*p* < 0.05). Consistent with this pattern, compliance with target time‐to‐care standards was significantly lower in the highest urgency levels (23.8% in Level I and 14.7% in Level II) and substantially higher in lower acuity categories (74.3%–90.4% in Levels III, IV and V).

In terms of care intensity, the need for re‐evaluation was higher in the most urgent levels (21.7% and 18.6% in Levels I and II, respectively) and decreased to 13.9% in Level V. The mean number of re‐evaluations remained relatively stable across the five priority levels, ranging from 1.12 to 1.30.

Triage duration was longer in Levels III and IV (39.42 ± 49.62 and 33.52 ± 39.08 s, respectively) and shorter in Level V (16.47 ± 34.51 s). ED LOS was longest in Levels II and III (5.04 ± 4.12 and 4.01 ± 3.42 h, respectively) and shortest in Level V (0.90 ± 2.13 h).

Finally, vital sign measurement was recorded more frequently in Levels II and III (temperature 74.0%/61.9%; blood pressure 80.2%/71.8%; heart rate 68.1%/65.0%), whereas other parameters such as oxygen saturation were documented less often, with proportions ranging from 1.4% to 13.2%. Table 3 presents care time metrics, triage target‐time compliance variables and clinical process indicators across the different assigned priority levels.

**TABLE 3:** Care times, compliance and clinical processes by triage priority level.

Variable	Triage priority level				
	Level I	Level II	Level III	Level IV	Level V
Minutes from triage to first physician record entry (mean ± SD)	38.00 ± 43.07	44.15 ± 49.16	49.72 ± 47.22^a^, ^b^	60.58 ± 52.28^a^, ^b^, ^c^	80.59 ± 84.80^a^, ^b^, ^c^, ^d^
Time‐target compliance (%)*	23.8	14.7	74.3	88.1	90.4
Need for re‐evaluation (%)*	21.7	18.6	17.2	10.3	13.9
Number of re‐evaluations (mean ± SD)	1.30 ± 1.16	1.23 ± 0.99	1.20 ± 0.51^b^	1.12 ± 0.39^a^, ^b^, ^c^	1.16 ± 0.46^a^, ^b^, ^c^, ^d^
Triage duration, seconds (mean ± SD)	20.66 ± 35.50	19.91 ± 36.04	39.42 ± 49.62^a^, ^b^	33.52 ± 39.08^a^, ^b^, ^c^	16.47 ± 34.51^b^, ^c^, ^d^
ED LOS, hours (mean ± SD)	2.57 ± 3.78	5.04 ± 4.12^a^	4.01 ± 3.42^a^, ^b^	1.93 ± 2.30^a^, ^b^, ^c^	0.90 ± 2.13^a^, ^b^, ^c^, ^d^
Temperature recorded (%)*	16.2	74.0	61.9	43.1	13.4
Blood pressure recorded (%)*	27.3	80.2	71.8	33.8	12.4
Heart rate recorded (%)*	26.5	68.1	65.0	31.2	10.5
Oxygen saturation recorded (%)*	5.2	12.9	13.2	3.3	1.4

Abbreviation: SD, standard deviation.

^a^ *p* < 0.05 versus Level I.

^b^ *p* < 0.05 versus Level II.

^c^ *p* < 0.05 versus Level III.

^d^ *p* < 0.05 versus Level IV (ANOVA post hoc comparisons).

* *p* < 0.05 for categorical variables (Chi‐square test).

### 3.4 Correlations Among Clinical Variables, Care Times and Re‐Evaluations

Regarding ED performance, age was associated with a longer ED LOS (*r* = 0.290, *p* < 0.001) and with lower oxygen saturation (*r* = −0.380, *p* < 0.001), suggesting that older patients tend to present with poorer respiratory profiles and require longer care processes.

In addition, lower values of oxygen saturation were associated with longer LOS (*r* = 0.211, *p* < 0.001) and a higher number of re‐evaluations (*r* = 0.111, *p* < 0.001), consistent with greater clinical complexity and the need for closer monitoring during the ED stay.

From a triage process perspective, triage duration was positively associated with the number of re‐evaluations (*r* = 0.202, *p* < 0.001). Haemodynamic variables also showed strong collinearity (SBP‐MAP *r* = 0.984, *p* < 0.001; DBP‐MAP *r* = 0.841, *p* < 0.001). Table 4 presents the Pearson correlation matrix for the analysed variables.

**TABLE 4:** Pearson correlation matrix between clinical variables, care times and number of re‐evaluations.

	Age	Temperature	SBP	DBP	MAP	HR	Oxygen saturation	Triage time	LOS	Time‐to‐first physician record entry	Number of re‐evaluations
Age	—	−0.101**	0.203**	−0.079**	0.108**	−0.122**	−0.380**	0.040**	0.290**	−0.056**	0.044**
Temperature		—	−0.084**	−0.077**	−0.089**	0.288**	−0.123**	0.004**	0.035**	0.005**	0.028**
SBP			—	0.632**	0.948**	−0.069**	0.051**	0.006**	−0.083**	0.015**	−0.024**
DBP				—	0.841**	0.137**	0.122**	0.000	−0.128**	0.014**	−0.024**
MAP					—	0.008**	0.086**	0.004*	−0.109**	0.016**	−0.026**
HR						—	−0.087**	0.004	0.041**	−0.034**	0.015**
Oxygen saturation							—	−0.024**	−0.211**	0.058**	−0.111**
Triage time								—	0.047**	−0.008**	0.202**
LOS									—	−0.162**	−0.071**
Time to first physician record entry										—	−0.011**
Number of re‐evaluations											—

* *p* < 0.05.

** *p* < 0.001.

### 3.5 Factors Associated With the Need for Re‐Evaluation

In the multivariable analysis, age remained associated with a higher likelihood of re‐evaluation, particularly in the 45–64 years (OR = 1.212; *p* = 0.016), 65–74 years (OR = 1.292; *p* < 0.001) and > 75 years (OR = 1.202; *p* = 0.021) groups, using patients < 18 years as the reference category. In addition, male sex was modestly but significantly associated with an increased likelihood of re‐evaluation (OR = 1.076, *p* < 0.001).

With respect to triage acuity, using Level I as the reference, Levels II and III showed a lower likelihood of re‐evaluation (OR = 0.585, *p* = 0.049; and OR = 0.546, *p* = 0.026, respectively).

Regarding clinical and operational variables, oxygen saturation was inversely associated with re‐evaluation (OR = 0.988; *p* < 0.001), indicating that lower oxygen saturation values were linked to a greater need for re‐evaluation. ED LOS was also associated with increased odds of re‐evaluation (OR = 1.015; *p* < 0.001), suggesting greater care complexity in episodes requiring re‐evaluation. Haemodynamic variables showed small but statistically significant associations: DBP was associated with slightly higher odds of re‐evaluation (OR = 1.004; *p* < 0.001), whereas MAP showed the opposite association, although the effect size was small (OR = 0.998; *p* = 0.010). Table 5 presents the univariable and multivariable logistic regression results for factors associated with the need for re‐evaluation.

**TABLE 5:** Univariable and multivariable logistic regression of factors associated with the need for re‐evaluation.

Variable	Need for re‐evaluation			
	Univariable logistic regression		Multivariable logistic regression	
	Crude OR [95% CI]	*p*	Adjusted OR [95% CI]	*p*
Age				
< 18 years	Reference		Reference	
18–44 years	1.096 [1.051–1.143]	< 0.001	1.046 [0.893–1.224]	0.579
45–64 years	1.364 [1.308–1.442]	< 0.001	1.212 [1.036–1.417]	0.016
65–74 years	1.596 [1.528–1.668]	< 0.001	1.292 [1.103–1.514]	< 0.001
> 75 years	1.720 [1.649–1.794]	< 0.001	1.202 [1.028–1.404]	0.021
Gender				
Male	1.078 [1.063–1.094]	< 0.001	1.076 [1.041–1.113]	< 0.001
Female	Reference		Reference	
Triage level				
Level I	Reference		Reference	
Level II	0.831 [0.672–1.027]	0.087	0.585 [0.343–0.988]	0.049
Level III	0.757 [0.614–0.934]	0.009	0.546 [0.321–0.931]	0.026
Level IV	0.420 [0.341–0.518]	< 0.001	0.601 [0.353–1.024]	0.061
Level V	5.126 [4.156–6.323]	< 0.001	1.066 [0.614–1.849]	0.820
DBP	1.002 [1.001–1.002]	< 0.001	1.004 [1.002–1.005]	< 0.001
MAP	1.002 [1.001–1.002]	< 0.001	0.998 [0.996–0.999]	0.010
Oxygen saturation	0.977 [0.974–0.979]	< 0.001	0.988 [0.985–0.992]	< 0.001
Triage time	1.007 [1.005–1.009]	< 0.001	0.996 [0.995–0.996]	< 0.001
ED LOS	1.066 [1.064–1.069]	< 0.001	1.015 [1.011–1.020]	< 0.001

*Note:* Nagelkerke's *R* ^2^ = 0.263.

Abbreviations: DBP, diastolic blood pressure; ED LOS, emergency department length of stay; MAP, mean arterial pressure; OR, odds ratio.

## 4 Discussion

In this analysis, care was predominantly provided to patients with intermediate and low acuity (mainly Levels III–IV), with vital signs consistent with the assigned urgency level and less favourable clinical profiles in the highest acuity categories. The interval between triage completion and the first physician record entry increased progressively as priority decreased, although compliance with target‐time thresholds was lower in Levels I–II. In addition, re‐evaluations were associated with older age, male sex, higher SBP and longer ED LOS, whereas priority Levels II–III, higher MBP, higher oxygen saturation and longer triage time were associated with a lower need for re‐evaluation.

Regarding STS priority levels, as in previous publications (Zachariasse et al. 2017; Montero Pérez et al. 2020), the highest frequencies in the present study were observed in the lowest acuity categories. Several factors may explain this pattern. First, ED access may be initiated by the patient based on a subjective perception of severity (Pérez‐Milena et al. 2023; Slagman et al. 2025). Other influential factors may include population income level, hospital complexity and difficulties obtaining timely outpatient medical appointments (Pérez‐Milena et al. 2023; Slagman et al. 2025; Mora et al. 2025).

In the present study, shorter time‐to‐care intervals were observed in higher triage priority levels; however, this variable did not influence the need for re‐evaluations. In this context, other studies (Wretborn et al. 2023; Nurchis et al. 2025; Mangino et al. 2024), consistent with our findings, have not identified time‐to‐physician targets as a major determinant. These shared results may be attributable to the study populations, which—similar to ours—were heterogeneous and not exclusively composed of patients with time‐dependent conditions (Wretborn et al. 2023; Nurchis et al. 2025; Mangino et al. 2024). Nevertheless, compliance with time‐to‐care targets was lower in priority Levels I and II, which contrasts with previous studies (Elkum et al. 2011; Bienzeisler et al. 2024) reporting shorter intervention times as triage priority increases. This discrepancy may be explained by the way time‐to‐care was estimated in our sample, using the time of medical record opening as the starting point; consequently, immediate patient care may have been prioritized over electronic documentation, a phenomenon also reported in several studies (Bienzeisler et al. 2024; Lauks et al. 2016).

Regarding triage duration, previous research (Zaboli, Brigo, Brigiari, et al. 2025; Bambi et al. 2016) has described shorter triage times in higher acuity levels. These findings are consistent with those observed in the present study. One possible explanation is that intermediate‐ and high‐priority levels, compared with immediate‐care or low‐priority categories, may more frequently require the measurement and confirmation of vital signs, thereby increasing the time spent in triage (Bambi et al. 2016).

These findings (Bambi et al. 2016) are robust when compared with those of the present analysis, as vital sign assessment in our study was more frequent in priority Levels II and III according to the STS. In contrast to our results, another study (Leow et al. 2025) reported lower rates of vital sign measurement in high‐acuity categories because immediate care is prioritized. However, this discrepancy may be explained by differences in the study population: that study examined a sample composed exclusively of patients with imminent cardiac arrest, a clinical condition with its own specific assessment (Leow et al. 2025) and that is consistently classified as maximum priority (Tsai et al. 2022; Mitchell et al. 2020).

Considering factors associated with the need for re‐evaluation in the ED, several studies (Wang et al. 2025; Tangkulpanich et al. 2021; Müller et al. 2024), in line with our findings, identify older age (Wang et al. 2025; Tangkulpanich et al. 2021; Müller et al. 2024) and oxygen saturation (Wang et al. 2025) as risk factors for requiring re‐evaluation, mainly due to the increased likelihood of clinical instability. These results are also consistent with the observation that blood pressure values influence subsequent ED flow, given their direct relationship with patient stability (Liu et al. 2025).

Another variable associated with the likelihood of subsequent re‐evaluation in the logistic regression was ED LOS, which is consistent with findings from a previous study (Tangkulpanich et al. 2021). In that study, longer LOS was directly associated with the need for ED revisits within 48 h (Tangkulpanich et al. 2021).

With respect to triage acuity, prior investigations (Kao et al. 2023; Sung et al. 2021) have reported that higher severity triage levels are associated with a greater need for re‐evaluation. These findings contrast with those of the present study, in which priority Levels II and III were associated with a lower likelihood of re‐evaluation compared with Level I. However, these differences may be attributable to variation in the triage system used, differences in the characteristics of the recruited samples (Kao et al. 2023; Sung et al. 2021) or the relatively low proportion of patients classified as Level I in our analysis.

### 4.1 Strengths and Limitations

Despite the analyses performed, this study has several limitations. First, its observational design precludes casual inference given that data were collected under real‐world clinical practice conditions rather than in an investigator‐controlled setting. In addition, as a single‐centre study, the generalizability of these findings to other ED settings should be interpreted with caution. Contextual variables such as ED crowding, staffing and workflow‐related factors were not available in the hospital dataset and therefore could not be analysed, although time to first medical evaluation may have been influenced by these external factors.

As key strengths, the large sample size increases the precision of the estimates and the statistical power to detect associations. In addition, inclusion of a complete 6‐year period enhances the representativeness of real‐world practice by capturing temporal variability (e.g., seasonality and changing demand scenarios). Overall, these findings provide useful information to inform quality‐improvement initiatives in the triage process and ED patient flow, particularly regarding operational aspects and nursing‐related documentation quality.

### 4.2 Implications for Clinical Practice

The present findings may contribute to emergency nursing practice by offering a pragmatic view of how triage‐related care processes are reflected in routine emergency care. The lower recorded compliance with target times in the highest acuity categories may reflect the prioritization of immediate clinical intervention over electronic documentation in critical situations. This highlights the importance of optimizing documentation workflows in high‐priority cases so that urgent care can be delivered without compromising the completeness and traceability of triage records. In parallel, the progressive differences observed in time to first physician record entry and ED LOS provide useful information on how priority stratification interacts with post‐triage patient flow in real‐world emergency care.

The findings related to re‐evaluation also support the need for closer post‐triage monitoring in selected patient profiles, particularly in older individuals and in those presenting less favourable physiological parameters. Although these associations should be interpreted cautiously, they may help ED teams identify patients who could benefit from earlier reassessment or escalation during their ED stay.

## 5 Conclusion

In conclusion, the nurse‐led STS demonstrated coherent clinical and operational stratification, with a predominance of low and intermediate acuity levels and less favourable physiological profiles in the highest urgency categories. In terms of patient flow, time to the first physician record entry and ED LOS varied consistently with acuity; however, lower compliance with target time thresholds in Levels I and II suggests a likely mismatch between immediate‐care delivery and its electronic documentation.

Finally, the need for re‐evaluation was mainly associated with older age, male sex, lower oxygen saturation and longer ED LOS, identifying higher complexity profiles that may benefit from closer monitoring during their ED stay. These findings support the use of nurse‐led STS as a prioritization tool in real‐world practice.

## Author Contributions

Conceptualization: D.S.‐M., J.A.L.‐A., V.S.‐F., J.R.‐S. Data curation: D.S.‐M., V.S.‐F. Formal analysis: D.S.‐M., V.S.‐F. Funding acquisition: Not applicable. Investigation: D.S.‐M., V.S.‐F., I.P.‐R. Methodology: D.S.‐M., V.S.‐F., I.P.‐R., C.M.G.‐A. Project administration: J.A.L.‐A., J.R.‐S., Á.L.‐G. Resources: J.R.S., Á.L.‐G., G.M.‐C. Software: V.S.‐F., J.D.F.‐S. Validation: D.S.‐M., J.A.L.‐A., V.S.‐F., J.M.C.‐T., Á.L.‐G., I.P.‐R., C.M.G.‐A., J.D.F.‐S., G.M.‐C., J.R.‐S. Visualization: V.S.‐F., C.M.G.‐A., J.D.F.‐S. Writing – original draft: D.S.‐M., V.S.‐F., J.D.F.‐S. Writing – review and editing: V.S.‐F., J.R.S., J.A.L.‐A., G.M.‐C.

## Funding

The authors have nothing to report.

## Disclosure

Compliance with the journal's statistical guidelines: The authors have checked to make sure that our submission conforms as applicable to the Journal's statistical guidelines.

## Ethics Statement

This study was approved on May 29, 2025, by the Research Ethics Committee for Medicinal Products at Albacete General Hospital with approval code: 2024‐096.

## Conflicts of Interest

The authors declare no conflicts of interest.

## Supporting information


**Appendix S1:** STROBE statement—checklist of items that should be included in reports of cohort studies.

## Data Availability

The data that support the findings of this study are available from the corresponding author upon reasonable request.
